# An Overview of HIV Prevention Interventions for People Who Inject Drugs in Tanzania

**DOI:** 10.1155/2013/183187

**Published:** 2013-01-03

**Authors:** Eric A. Ratliff, Sheryl A. McCurdy, Jessie K. K. Mbwambo, Barrot H. Lambdin, Ancella Voets, Sandrine Pont, Haruka Maruyama, Gad P. Kilonzo

**Affiliations:** ^1^Division of Health Promotion and Behavioral Sciences, University of Texas School of Public Health, University of Texas Health Science Center at Houston (UTHealth), 26th Floor, 7000 Fannin Street, Houston, TX 77030, USA; ^2^Department of Psychiatry and Mental Health, Muhimbili National Hospital, Dar es Salaam, Tanzania; ^3^Department of Global Health, University of Washington, Seattle, WA 98104, USA; ^4^Pangaea Global AIDS Foundation, 472 Ninth Street, Oakland, CA 94607, USA; ^5^Médecins du Monde, P.O. Box 105948, Dar es Salaam, Tanzania; ^6^Muhimbili University of Health and Allied Sciences, P.O. Box 65466, Dar es Salaam, Tanzania

## Abstract

In the past decade, Tanzania has seen a rapid rise in the number of people who inject drugs (PWID), specifically heroin. While the overall HIV prevalence in Tanzania has declined recently to 5.6%, in 2009, the HIV prevalence among PWID remains alarmingly high at 35%. In this paper, we describe how the Tanzania AIDS Prevention Program (TAPP), Médecins du Monde France (MdM-F), and other organisations have been at the forefront of addressing this public health issue in Africa, implementing a wide array of harm reduction interventions including medication-assisted treatment (MAT), needle and syringe programs (NSP), and “sober houses” for residential treatment in the capital, Dar es Salaam, and in Zanzibar. Looking toward the future, we discuss the need to (1) *extend* existing services and programs to reach more PWID and others at risk for HIV, (2) *develop* additional programs to strengthen existing programs, and (3) *expand* activities to include structural interventions to address vulnerabilities that increase HIV risk for all Tanzanians.

## 1. Introduction

Until recently, heroin use and associated health risks were not perceived as major issues in sub-Saharan Africa. However, in the mid-1980s and early 1990s, East Africa became an important stop along international drug trafficking routes thereby introducing heroin in the region [1]. Consequently, heroin injection emerged in Dar es Salaam, Tanzania, in the late 1990s [2, 3]. The Tanzanian government acknowledges that injection drug use is a well-established problem across many cities in the country [4]. 

During the 2000s, the rapid escalation of injection drug use in Tanzania accompanied a synergistic rise in HIV prevalence among people who inject drugs (PWID) due to unsafe injection practices and risky sexual behavior. Since 2003, the majority of PWID in Dar es Salaam reported injecting three times per day [2, 5, 6], with 41% of all users sharing needles in the past 30 days [6]. In addition, 28% of PWID reported reusing used rinse water [1], which is not effective in preventing HIV. With regard to sexual risk behavior, only 42% of PWID reported using condoms during sex in the last 30 days [7]. Studies conducted between 2003 and 2007 estimated an HIV prevalence of 42% among PWID [6, 8], compared to an estimated prevalence of 9% in the general population [9]. Subsequent studies conducted between 2009 and 2010 found an HIV prevalence among younger PWID of approximately 31% and 35%, respectively [5, 10]. These studies also found a much higher prevalence among women who inject drugs from 55% to 68%, respectively. Although overall HIV prevalence rates in Tanzania have declined from 8.7% in 1999 to 5.6% in 2009 [9, 11], continuing high rates of PWID-related HIV infection threaten to destabilize the progress that Tanzania has made in controlling its HIV epidemic. The importance of addressing the high HIV burden among PWID is critically important in its own right and is especially so because they often engage in risky sexual behaviors with people who do not inject drugs. Neglecting HIV among PWID could spur a resurgence of HIV throughout the Tanzanian population.

## 2. Harm Reduction in Tanzania

On World AIDS Day 2006, researchers from Muhimbili University of Health and Allied Sciences (MUHAS) and the University of Texas School of Public Health (UT-Houston) conducted a day-long media workshop in Dar es Salaam to present findings they had published in peer-reviewed journals to the press, community-based organisations (CBOs), nongovernmental organisations (NGOs), and donors. The national press coverage of the event and of other antidrug trafficking efforts presaged the Tanzanian government's 2007 request to direct some funds from the President's Emergency Funds for AIDS Relief (PEPFAR) toward HIV prevention outreach projects specifically targeting PWID. 

In Dar es Salaam, PEPFAR funded the Tanzania AIDS Prevention Program (TAPP) in 2007 to implement prevention and outreach activities among PWID. TAPP is a consortium that MUHAS manages in partnership with UT-Houston, Pangaea Global AIDS Foundation (Pangaea), the Tanzanian Ministry of Health and Social Welfare (MoHSW), and the Tanzanian Drug Control Commission (DCC). TAPP partners coordinate a multiarm approach to HIV prevention and outreach, covering a broad population of clients. For the general population in Dar es Salaam, TAPP offers HIV testing and counseling (HTC) for individuals and couples through mobile van outreach, provider-initiated testing and counseling (PITC) and onsite testing at Muhimbili National Hospital, as well as training for health care personnel in prevention and outreach procedures. TAPP staff have also provided HTC services at annual events, including World AIDS Day, Africa Day, Rotary Club Day at Chanika, International Day Against Drug Abuse and Illicit Trafficking, and the *Saba Saba* Trade Fair. 

For PWID, the community-based outreach project helps to coordinate many intervention programs through the cooperation and input from local partner CBOs: Kimara Peer Educators and Health Promoters Trust Fund (KPE); Youth Volunteers Against Risky Behaviours (YOVARIBE); Centre for Human Rights Promotion (CHRP); and Blue Cross Society of Tanzania (BCST). These CBOs and two mobile clinics provide HTC outreach services for IDUs in the Kinondoni District of Dar es Salaam. These services include distribution of health kits (containing information, education, and communication (IEC) materials along with kits for wound care, condoms, and bleach kits for cleaning of works), psychosocial support services, individual counseling, self-support groups, Narcotics Anonymous (NA) and Methadone Anonymous (MA) meetings, and family group therapy meetings. As part of this project, UT-Houston, together with community outreach workers from MUHAS and CBO partners, maps communities to identify suitable locations for mobile units, and monitors current heroin use patterns in Dar es Salaam to modify program strategies. Additionally, TAPP provides continuing education training for community outreach workers. Through August 2012, TAPP partner CBOs have engaged over 11,800 heroin users through outreach activities, of which 2530 were PWIDs and referred 571 to the medication-assisted treatment (MAT) clinic (see below) [12, 13]. 

During February 2011, a dedicated public methadone-based MAT clinic, the second in sub-Saharan Africa after Mauritius, opened at the Muhimbili National Hospital. The MAT clinic is staffed year-round to accommodate the project participants who are all required to travel to the clinic on a daily basis to receive their dose of liquid methadone where consumption is directly observed by healthcare providers. CBO outreach efforts also include identifying, screening, and preparing possible candidates for medically assisted treatment. Through the development of service provision guides and clinical mentoring, Pangaea assisted in building local capacity to launch the MAT clinic and continues to support the services with ongoing clinical mentoring and the use of implementation science methodologies. From its opening to August 2012, TAPP has enrolled over 431 PWID in methadone treatment at its MAT clinic [13].

During late 2010, Médecins du Monde France (MdM-F) began a harm reduction program in the Temeke District of Dar es Salaam with a drop-in center (DIC) and outreach activities. This program is funded by the French Agency for Development (AFD), the Municipality of Paris, and private donors. In November 2010, MdM-F signed a Memorandum of Cooperation with the MoHSW and the district council in Temeke to strengthen local capacities of stakeholders in harm reduction. Between August 2011 and March 2012, MdM-F had engaged 2932 PWID and 1265 PWUD (people who use drugs, including those who do not inject) through outreach activities and the DIC. Approximately 45 PWID attend the DIC daily, and an additional 120 PWUD attend during the two days per week when the DIC is open to those who do not inject heroin. In March 2011, MdM-F began a needle and syringe program (NSP). This program distributes approximately 25,000 syringes and condoms per month, and tests clients for HIV, hepatitis B, and hepatitis C.

MdM-F also has special services for women due to their increased vulnerability as PWID/PWUD. There is a separate room for women at the DIC, and, once a week, the DIC is open only to women. Many bring their children as the DIC provides food assistance, and MdM-F provides a staff member to accompany pregnant clients to HIV and antenatal care at other clinics. MdM-F supports several self-support groups: one for women who use drugs, one for PWUD who are HIV-positive, and one for PWID enrolled in the MAT program. MdM-F has also been working with government and private clinics in Temeke to provide services that are more welcoming to PWID/PWUD and others who often experience discrimination in these settings (i.e., men who have sex with men, sex workers). Finally, because PWID/PWUD are often blamed for social problems and criminal activities, MdM-F advocates for fair treatment of PWID/PWUD in accordance with local laws.

To build local capacity and sustainability of harm reduction activities, MdM-F has initiated a “Temeke committee” to help design and expand interventions for PWUD. The committee coordinates government agencies and nongovernment organisations to strengthen referral systems, to exchange experiences and to advocate jointly at national level for increased attention and funding for HIV-prevention services for PWUD. MdM-F offers training and coaching to stakeholders who have a role in HIV prevention among PWUD in Temeke, including medical staff, police officers, community, and religious leaders. They also provide intensive training and mentoring through internships, exchange of staff and continuous consultation to several national nongovernment organisations: Poverty Fighters, Tayohag, and Mukikute. MdM-F has also been working with the Alliance of Mayors and Municipal Leaders on HIV/AIDS in Tanzania (AMICALL) to support the development of a strategic plan for HIV prevention efforts, improving access to care and treatment. For their PWID clients, MdM-F provides training and support for income-generating activities. Some have been trained as peer educators; others have received training and legal assistance to develop small businesses.

In Zanzibar, PEPFAR funded two projects to address the needs of PWID: The American International Health Alliance (AIHA) partnered with the DCC, MOHSW, and the Department of Substance Abuse Prevention and Rehabilitation (DSAPR) in Zanzibar, and the Great Lakes Addiction Technologies Transfer Center, based in Detroit, Michigan, to introduce the recovery oriented systems of care (ROSC) model in Zanzibar. These initial efforts focused on establishing recovery groups like Narcotics Anonymous, using the “12 Steps Recovery Model” and the “Islamic *Milati*.” A key PWID leader emerged from within this Zanzibari recovery group movement, Sulieman Mauly, and in 2009 through his CBO and with support from Bi Fatma and others he created the first Zanzibari “sober house” based on the 12-step recovery model. The program integrates group counseling with art therapy, yoga, and acupuncture. The men also play soccer and engage in meditation, and they can receive training in carpentry. By May 2012, there were nine sober houses in Zanzibar, including one for women that opened in 2010 [14]. 

The other PEPFAR-funded project on Zanzibar is United for Risk Reduction and HIV/AIDS Prevention (URRAP) initiated through Columbia University's International for AIDS Care and Treatment Programs (ICAP). URRAP involves a partnership between national government agencies and three nongovernmental organisations: Zanzibar Youth Education, Environment and Development Support Association; Zanzibar Association of Information against Drug Abuse and Alcohol; and the Zanzibar Youth Forum. Outreach workers provide PWUD with information regarding HIV prevention, linkage to HIV testing and, for those found to be HIV-infected, access to care and treatment. URRAP provides static and mobile HTC outreach services in conjunction with sexually transmitted infection (STI) and tuberculosis (TB) screening, and peer escort referrals for services and for those needing HIV care and treatment. Between January 2009 and March 2011, 698 PWID received HTC services in Zanzibar. Eighty-seven were HIV positive and 64 enrolled in care and treatment [15].

## 3. Improving HIV Prevention among PWID

TAPP, MdM-F, and other organisations have made great strides in providing behavioral interventions to prevent the spread of HIV among PWID in Tanzania. Now that these organisations have created the basic structures and established the viability of medication-assisted treatment (MAT), needle and syringe programs (NSP), and other forms of drug treatment and rehabilitation, there is a need to improve the implementation of HIV prevention interventions along three dimensions: (1) *extend* existing services and programs to reach more PWID and others at risk for HIV; (2) *develop* additional programs to strengthen existing programs; and (3) *expand* activities to include structural interventions to address vulnerabilities that increase HIV risk for all Tanzanians.

### 3.1. Extending Existing Services

Extending existing services can occur along several distinct avenues. First, harm reduction organisations are extending services geographically to reach a greater number of PWID. To date, interventions to reduce HIV risk behaviors among PWID in Tanzania are limited to Zanzibar and parts of the capital, Dar es Salaam. TAPP, with funding from the Open Society Foundation, is preparing to launch a needle and syringe program (NSP) in Kinondoni District of Dar es Salaam during 2012 to complement MDM-F's existing NSP activities in the neighboring Temeke District. TAPP is also extending HIV testing and counseling (HTC) services and the recruitment of PWID for the MAT program to the other two districts of Dar es Salaam (Ilala and Temeke) and is exploring different options for distribution of methadone beyond the clinic at Muhimbili National Hospital. As part of this effort, MdM-F has recently begun referring PWID clients to MAT clinic at Muhimbili Hospital. The American International Health Alliance (AIHA) team from Zanzibar, with assistance from the Detroit Recovery Program, is expanding the 12-step program and sober houses to Dar es Salaam and other areas of mainland Tanzania. Efforts are also underway to extend existing programs and services to PWID across the country. TAPP is collecting behavioral data and estimating PWID population sizes in the cities of Arusha and Tanga for possible extension of intervention programs, and other organisations are conducting similar surveys in Mbeya, Mwanza, Iringa, and other towns with an eye toward implementing similar programs for PWID in those areas.

Second, there is a strong argument for extending drug treatment services to noninjecting heroin users. Most heroin users in Tanzania consume the drug by smoking it in a “cocktail” (*kokteli*) of tobacco and marijuana. For those who transition from smoking to injecting, the median transition time from smoking to injection is five years, and those PWUD under 25 years of age transition in two years [10]. TAPP and its partner CBOs currently provide outreach services including education, HIV testing, condom distribution, and facilitating Narcotics Anonymous meetings for heroin smokers in the target area. However, those who smoke heroin are not eligible for MAT services due to limited resources. There is a programmatic rationale for targeting PWID first because their injection practices place them at increased risk for contracting and transmitting HIV to others. But this is also a compelling reason for preventing smokers from transitioning to riskier injecting practices [16], and research has shown that noninjecting heroin users exposed to treatment (e.g., residential detoxification, methadone maintenance, and outpatient rehabilitation) are less likely to initiate injecting [17].

Third, extending services also entails addressing PWIDs' risky behaviors that might be associated with other targeted populations. There is a need to coordinate and integrate programs for people who are categorized as members of different key populations (KPs)—including PWID, men who have sex with men, and sex workers—as their risky behaviors often overlap [12]. For example, most Tanzanian women who inject heroin also engage in sex work, and more than half of the MSM sampled in a recent study had been married or cohabited with women [18]. Yet we in public health often approach people who engage in these behaviors as distinct “risk groups,” and develop intervention programs that emphasize the primary behavior for the targeted group. Local organisations often follow this logic, focusing on a particular group without addressing how these risky behaviors intersect. Using the targeted approach, we overlook people at risk who do not fit into our narrow objectives and we do not use our limited resources as efficiently as we could. By integrating intervention activities to cover all risky behaviors instead of focusing on certain groups, we could move away from the outdated “risk group” approach, reducing stigmatization and discrimination in the process. TAPP and MdM-F recognize the importance of integrating services originally designed for different KPs, and are developing programs to address these intersecting behaviors and identities (see below).

### 3.2. Developing Additional Programs

In terms of developing additional programs, MdM-F has been advocating a comprehensive approach to harm reduction as defined by various UN agencies (WHO/UNAIDS/UNODC). In Temeke, MdM-F strives to offer a holistic package of interventions, including psychosocial support, economic (re)integration, overdose prevention and management, and defense of human rights for PWID/PWUD. TAPP is also planning a broad array of intervention activities and services to enhance the efficacy of existing programs. First, because HIV is spread through intimate activities such as sexual intercourse and needle/syringe sharing, TAPP is developing programs that move beyond individual behaviors to address these intimate interactions, including (a) targeting couples for testing, counseling, and other intervention activities and (b) integrating gender-based and interpersonal violence reduction strategies into counseling and outreach activities. In addition to adapting and developing the content for these activities, TAPP will train partner CBOs and service providers from government ministries (e.g., MOHSW) to implement these interventions.

Second, because of the synergistic intersections between polysubstance abuse, violence, and risky sexual behavior, TAPP is developing a brief, motivational intervention to reduce alcohol consumption among PWID and others who use TAPP services. TAPP will also integrate alcohol moderation messages into community outreach activities. Third, TAPP is also exploring the possibilities for more women-friendly services, such as extending MAT clinic hours and locations for those PWID who are sex workers and unable to travel to the MAT clinic during daytime hours.

Finally, TAPP is developing additional clinical services to address comorbidities among PWID and members of other key groups. These clinical services include (a) integrated HIV and TB testing and treatment, and testing for hepatitis B and C; (b) training of “treatment navigators” to assist clients in their use of health and social services; and (c) training of local clinic and organisation personnel in issues of stigma, human rights, and values clarification concerning sexual orientation, drug use, and sex work, as well as awareness of specific health needs of people who engage in these activities [19].

### 3.3. Expanding Activities to Include Structural Interventions

While behavioral interventions (e.g., HTC, MAT, and NSP) have proven effective in reducing HIV/AIDS prevalence among PWID in high-income countries, many countries in sub-Saharan Africa face structural challenges that need to be addressed before long-term, sustainable changes can occur. Many of the drug users in Dar es Salaam use heroin to escape the harsh realities of life associated with past traumas and continuing limitations on political and economic opportunities [20]. While this is true in high-income countries too, in the Tanzanian setting it is complicated by the lack of support many PWID experience as orphans. 

For example, sixty-eight percent of young PWID in a 2009-2010 survey were orphaned [20]. The experience of a PWID epidemic emerging in the third and fourth decades of the AIDS epidemic in Africa reveals deep chasms in the social support systems upon which communities traditionally relied. The trauma of parental loss and lack of access to a range of resources exists within a framework of other limitations originating in underlying social structures that people in power use to foster discrimination, inequity, and exclusion. Social structures are manifestations of social norms, where people use difference—in gender, sexuality, religion, economic status, political affiliation, and so forth—to express power and control over one another. Social structures include class and gender schemes that privilege one group over another; social structures also include formal institutions such as legal frameworks that criminalize certain behaviors (drug use, prostitution, homosexuality, etc.). These structures thus increase the vulnerability of people who are marginalized because they do not always follow social norms and lack strong family support, predisposing them to more harmful behaviors and outcomes through discrimination and hampering their ability to change their lives [21]. So even if TAPP's current harm reduction activities are successful in reducing or eliminating heroin dependence among individual PWID, many will continue to face discrimination that diminishes their long-term prospects, making it more difficult for them to fully recover from their addiction.

 These social structures exist across different levels of society (family, community, nation) and interact with biological organisms and individual behaviors to form a complex, dynamic system. Because these systems are composed of numerous elements and change in unpredictable (nonlinear) ways, a comprehensive approach using a variety of methodologies is required. “Combination prevention” includes biomedical, behavioral, and structural interventions for HIV prevention tailored to local conditions [22, 23]. TAPP is working with ministries at the national level (e.g., DCC and MOHSW) to advocate for changes in policies and practices that can reduce the vulnerability of PWID and other key populations. Moreover, their partner CBOs work closely with government officials at the local level to coordinate outreach activities, identify additional resources, and assist harm reduction clients. 

MdM-F is also developing programs to help vulnerable communities speak for themselves. In collaboration with Openair Communication, MdM-F has trained a group of PWUD to use film and music as advocacy tools. For World Aids Day 2011, clients produced the music video *Inawezakana*, calling for an end to the HIV epidemic in the country through harm reduction services. In August 2012, group screened its first short film *Mdudu Mbaya* (literally “bad insect” in Swahili, but also a euphemism for “bad virus”). These productions are widely distributed to raise awareness throughout Tanzanian society and to foster a sense that everyone in Tanzania is addressing HIV together. The group is also planning to produce an educational video on HIV prevention. Along the same line the initiatives mentioned earlier (peer educators, support groups, creation of local and national PWUD-networks) are strongly supported by MdM-F.

While these organisations have made great strides in promoting individual harm reduction, more work on these structural barriers remains, and every harm reduction organisation is looking at new ways to integrate different interventions at various levels to ensure that behavioral interventions produce better outcomes and are sustainable beyond the life of the programs. Unless organisations can coordinate with governments, other social institutions, and communities to address structural disparities, help recovered users reintegrate into communities and pursue functional positions in society, harm reduction may only offer short-term, temporary benefits. TAPP, MdM-F, and the other organisations are committed to responding to the large numbers of PWID and their families and communities, but coordination with government and nongovernment organisations is needed to address the many contentious and complex issues associated with heroin use in Tanzania. 

## 4. Conclusion

TAPP, MdM-F, and the other organisations working in Dar es Salaam and Zanzibar are pioneers of harm reduction in sub-Saharan Africa because we have overcome considerable ideological and structural barriers to offer services such as medication-assisted treatment and needle and syringe programs. These are monumental achievements given a political atmosphere that favors drug trafficking control and the criminalization of drug use. Furthermore, the Tanzanian government has promised to continue supporting clients in the MAT program if external funding ends, signifying a long-term commitment to recognizing and addressing PWID needs. Tanzania is one of only two countries in sub-Saharan Africa to offer public methadone services, and one of the few countries to have recognized the severity of HIV in the PWID population. Government agencies, community-based organisations, and international collaborators have come together to respond to the problem via specific public health programming. Looking toward the future, organisations working with PWID/PWUD should strive to integrate input from the community during program planning stages to develop well-targeted, streamlined programs that address the specific experiences and needs of that community. Harm reduction organisations working in Tanzania continue to develop collaborative efforts to serve PWID/PWUD, but we need strong support from national governments and regional intergovernmental organisations such as the East African Community (EAC). While there are many challenges in the road ahead, these harm reduction programs serve as positive examples for the possibility of coordinated public health responses to PWID epidemics in sub-Saharan Africa.
